# mTOR Signaling, Translational Control, and the Circadian Clock

**DOI:** 10.3389/fgene.2018.00367

**Published:** 2018-09-10

**Authors:** Ruifeng Cao

**Affiliations:** ^1^Department of Biomedical Sciences, University of Minnesota Medical School, Duluth, MN, United States; ^2^Department of Neuroscience, University of Minnesota Medical School, Minneapolis, MN, United States

**Keywords:** mRNA, translational control, circadian clock, mTOR, entrainment, synchronization, oscillation, SCN

## Abstract

Almost all cellular processes are regulated by the approximately 24 h rhythms that are endogenously driven by the circadian clock. mRNA translation, as the most energy consuming step in gene expression, is temporally controlled by circadian rhythms. Recent research has uncovered key mechanisms of translational control that are orchestrated by circadian rhythmicity and in turn feed back to the clock machinery to maintain robustness and accuracy of circadian timekeeping. Here I review recent progress in our understanding of translation control mechanisms in the circadian clock, focusing on a role for the mammalian/mechanistic target of rapamycin (mTOR) signaling pathway in modulating entrainment, synchronization and autonomous oscillation of circadian clocks. I also discuss the relevance of circadian mTOR functions in disease.

## Introduction

As explained by the central dogma of molecular biology, genetic information flows from DNA to RNA to make a functional product, a protein. Protein synthesis accounts for ∼75% of a cell’s total energy consumption and is highly regulated in cells (Lane and Martin, 2010). Translational control (regulation of protein synthesis) plays a significant role in the regulation of gene expression under physiological conditions. Deregulation of translational control is frequently involved in the pathophysiology of human diseases, including cancer, metabolic syndromes, and neurological disorders (Sonenberg and Hinnebusch, 2007; Hershey et al., 2012).

Circadian (∼24 h) rhythmicity is an autonomous biological property that controls a variety of biochemical, physiological, and behavioral processes in all living organisms (Hall and Rosbash, 1993; Young, 1998; Takahashi et al., 2008). The rhythmic processes are driven by autonomous oscillations of “clock genes” in cells. Whereas a significant role for protein synthesis in the circadian clock was found half a century ago (Feldman, 1967, 1968), novel mechanisms of mRNA translation control are being discovered in recent years. Some of these findings have been nicely summarized in three reviews (Lim and Allada, 2013b; Green, 2018; Torres et al., 2018). Here I discuss the latest progress in our understanding of translational control mechanisms in the circadian clock, focusing on a critical role for the mammalian/mechanistic target of rapamycin (mTOR) signaling pathway.

## Circadian Rhythms and Circadian Clocks

Circadian rhythms are endogenously driven by proteins called “circadian clocks” that oscillate in either their physical levels or functional states on a daily basis. The fundamental property enables organisms to temporally coordinate their physiology and behavior, according to changes in daily light/darkness cycles, food availability, temperature, moisture, and air pressure in the environment (Rosbash, 2009). Thus, organisms can predict and prepare for upcoming environmental changes to meet their physiological needs (Reppert and Weaver, 2002). Rhythmic physiological and metabolic processes are normally coupled and synchronized to the environmental cycles so that optimal physiological and metabolic efficiencies can be attained at the right time of a day.

The circadian system is hierarchically organized. In *Drosophila* the central clock cells are located in the large and small lateral ventral neurons (l-LNvs and s-LNvs) of the optic lobe (Dubowy and Sehgal, 2017), which synthesize pigment dispersing factor (PDF) as a circadian neuromodulator among clock neurons. In mammals, the suprachiasmatic nucleus (SCN) of the anterior hypothalamus is the master pacemaker (Moore, 2013). SCN receives photic input from the retina, generates robust circadian rhythms and sends out neural and endocrine signals as rhythmic outputs to various brain regions as well as peripheral organs and systems. In the body, functions of the autonomic nervous system, endocrine and immune systems are all regulated by the SCN (Mohawk et al., 2012; Gamble et al., 2014). Clock genes are ubiquitously expressed in almost all cells and tissues. Almost all types of cells can perform circadian oscillations, with different robustness, accuracy, and period (Liu et al., 2007). Thus, rhythms in various organs and systems need to be orchestrated by the master pacemaker and synchronized to the environmental light/dark cycles (Aton and Herzog, 2005; Golombek and Rosenstein, 2010).

In cells circadian oscillations are driven by autonomous genetic feedback loops. Work over past three decades has identified evolutionarily conserved transcriptional/translational feedback loops (TTFLs) and about a dozen genes that account for cellular circadian oscillations (Hall and Rosbash, 1993; Young, 1998; Takahashi, 2017). In mammals, the heterodimers of transcription factors CLOCK and BMAL1 activate gene transcription of *Per* and C*ryptochrome* (*Cry*). PER and CRY proteins form multiprotein complexes. Once the complexes accumulate to certain levels in the cytosol, they translocate back to the cell nucleus, associate with CLOCK/BMAL1 heterodimers, and repress *Per* and *Cry* gene transcription (Takahashi et al., 2008).

*Per* gene expression functions as a “knob” of the clock and is tightly regulated by intracellular and extracellular signals via complex mechanisms. Firstly, rhythmic *Per* transcription is activated by the CLOCK: BMAL1 complexes through the E-box enhancers in the promoter region. Secondly, at the post-transcriptional level *Per* mRNA processing is regulated by methylation (Fustin et al., 2013). Thirdly, as the degradation rate of PER proteins is also a key determinant of the length of a circadian cycle, PER cycling is controlled by sophisticated post-translational modifications such as phosphorylation (Kloss et al., 1998; Lowrey et al., 2000; Lee et al., 2001; Meng et al., 2008; Chiu et al., 2011) and ubiquitination (Busino et al., 2007; Siepka et al., 2007; Hirano et al., 2013; Yoo et al., 2013).

Recent work has started to uncover a key role for translational control in regulating clock gene expression. In *Drosophila*, the RNA binding proteins Ataxin-2 (Atx2) interacts with Twenty-four (Tyf) to activate *Per* mRNA translation in pacemaker neurons to sustain robustness of circadian behavioral rhythms (Lim et al., 2011; Lim and Allada, 2013a; Zhang et al., 2013). A targeted RNAi screen revealed knockdown of the atypical translation factor NAT1 lengthens circadian period and reduces PER protein levels in PDF neurons (Bradley et al., 2012). In mice, we show that as the downstream targets of the mitogen-activated protein kinase (MAPK)/extracellular Signal-regulated Kinase (ERK) pathway, MAPK interacting protein kinases (MNKs) phosphorylate the cap-binding protein eIF4E in the SCN. Activities of the MAPK/MNK/eIF4E pathway can be activated upon light exposure at night. Phosphorylation of eIF4E stimulates *Per1* and *Per2* mRNA translation and functions as a facilitator of photic entrainment of the SCN circadian clock (Cao et al., 2015). Besides these mechanisms, another emerging translational control pathway with more complexity is the mTOR signaling.

## mTor Signaling

mTOR is an evolutionarily conserved serine/threonine protein kinase, also known as FK506-binding protein 12-rapamycin-associated protein 1 (FRAP1). mTOR forms two multiprotein complexes in cells, the mTOR complex (mTORC) 1 and mTORC2. mTORC1 and mTORC2 share some protein components, including mTOR, mLST8 (mammalian lethal with sec13 protein 8, also known as GβL), and DEPTOR (the inhibitory DEP domain containing mTOR-interacting protein). mTORC1 also includes Raptor (the regulator-associated protein of the mammalian target of rapamycin) and PRAS40 (proline-rich Akt substrate of 40 kDa). Raptor interacts with the TOS (target of rapamycin signaling) motifs mTOR in a rapamycin-sensitive manner and is essential for mTORC1 activity. mTORC2 consists of Rictor (the rapamycin insensitive companion of mTOR), mSIN1(mammalian stress activated MAP kinase-interacting protein 1), and PROTOR 1 and 2 (proteins observed with rictor 1 and 2). Rictor and mSIN1 are both critical for mTORC2 function (Lipton and Sahin, 2014; González and Hall, 2017; Saxton and Sabatini, 2017).

mTOR signaling refers to an intracellular signaling network centered on mTORC1 and mTORC2. mTOR signaling senses intracellular signals and also responds to extracellular stimuli. It can be activated by upstream signals including growth factors (e.g., insulin and insulin-like growth factor-1), energy status (e.g., oxygen and ATP levels), nutrients (e.g., leucine and arginine), as well as neurotransmitters (e.g., glutamate and neuropeptides). Growth factors and mitogens inhibit the Tuberous Sclerosis Complex (TSC) complex. TSC is a key negative regulator of mTORC1. It is a GTPase activating protein for the small GTPase Rheb, which directly binds and activates mTORC1. Once activated, mTOR signaling controls fundamental biological processes including protein synthesis and turnover, lipid and glucose metabolism, autophagy, cytoskeleton organization, etc. (González and Hall, 2017; Saxton and Sabatini, 2017). mTORC1 has the most defined role in translational control. mTORC1 exhibits protein kinase activity and regulates mRNA translation by regulation of its translation effectors, which include the eukaryotic initiation factor 4E-binding proteins (4E-BPs) and ribosomal protein S6 kinases (S6K1 and S6K2) (Hay and Sonenberg, 2004).

## mTor and Translational Control

In general, translational control can be achieved via two mechanisms: (1) impacting on the mRNAs by sequence specific RNA binding proteins or small non-coding RNAs such as microRNAs; (2) impacting on the translational apparatus, which include translation factors, ribosomes and tRNAs. The latter predominantly affects the step of translation initiation (Hershey et al., 2012).

All nuclear transcribed mRNAs are capped at the 5′-ends with the 7-methyl-guanosine. Eukaryotic translation initiation factor 4E (eIF4E) is a cap-binding protein. It recognizes and binds to the mRNA 5′ m7GpppN (where N is any nucleotide) (Hinnebusch et al., 2016). eIF4G is a scaffolding protein that associates with 4E and 4A. eIF4A is a RNA helicase that resolves mRNA secondary structures. eIF4F (including 4E, 4G, and 4A) complex interacts with eIF3 to recruit the small ribosomal subunit and initiates cap-dependent translation initiation. The eIF4E-binding proteins (4E-BPs) control eIF4E binding to the cap structure. 4E-BP binding of eIF4E causes repression of cap-dependent translation initiation and can be relieved by phosphorylation of 4E-BPs through mTORC1 (Gingras et al., 1999). Activated by various extracellular and intracellular cues, mTORC1 phosphorylates 4E-BPs to lead to its dissociation from eIF4E (Brunn et al., 1997; Gingras et al., 1999), which allows cap-dependent mRNA translation to initiate. Thus, mTORC1 regulates cap-dependent translation via 4E-BPs.

As another major branch of mTORC1, S6K1 is activated by phosphorylation on its hydrophobic motif site, Thr389. S6K1 in turn phosphorylates a number of proteins that control mRNA translation. It phosphorylates eukaryotic translation initiation factor 4B (eIF4B) at S422, which is a cofactor of eIF4A and increases its processivity (Holz et al., 2005). S6K1 also phosphorylates and promotes the degradation of PDCD4 (Programmed Cell Death 4). PDCD4 inhibits eIF4B and enhances the translation efficiency of spliced mRNAs via its interaction with SKAR (S6K1 Aly/REF-like target, Dorrello et al., 2006), a component of exon-junction complexes involved in mRNA splicing (Ma et al., 2008). S6K1 inactivates eukaryotic elongation factor-2 kinase (eEF2K) (Wang et al., 2001; Knebel et al., 2002), which is a negative regulator of eukaryotic elongation factor 2 (eEF2), by phosphorylating it at S366, and thus regulates translation elongation.

## mTor and the Circadian Clock

Intracellular signal transduction pathways control circadian timing and entrainment by regulating clock gene expression at different levels (Gillette and Mitchell, 2002; Evans, 2016). In general, a signaling pathway that is important for the circadian clock is usually regulated by circadian rhythmicity and therefore exhibits rhythmic activities under constant conditions. Moreover, the signaling pathway is often regulated by the extracellular signals and couples these signals to circadian gene expression. Thus, the feedback loops within the clock is coupled to a feedforward loop involving the environmental cues, the signaling pathway and the clock. The mTOR pathway is a typical signaling example that couples environment cues to the clock cells and its network. In this section, I will discuss the interactions between the mTOR pathway and the circadian clock.

### mTOR Regulation of the Circadian Clock

Work over the past decade has started to uncover a multifaceted role of mTOR in the circadian clock. Firstly, mTOR signaling is part of the photic entrainment pathway in the SCN; secondly, mTOR regulates autonomous clock properties in a variety of circadian oscillators; thirdly, mTOR regulates network properties of coupled circadian oscillators, such as the SCN neurons.

#### Regulation of Photic Entrainment of the SCN Circadian Clock by mTORC1

To adapt to the changing environment, the circadian clock is constantly adjusted by environment signals. Light is the most important cue to regulate the SCN clock. Photic input is received by the retina and relayed to the SCN via the retinohypothalamic tract (RHT). The pathway is distinct from the image-forming visual pathway in that the reception of light is mediated by intrinsically photosensitive retina ganglion cells (ipRGCs), which express the photopigment melanopsin (Peirson and Foster, 2006; Panda, 2007). RHT terminals form synaptic connections with the ventral SCN neurons. The excitatory neurotransmitter glutamate and the neuropeptide pituitary adenylate cyclase-activating peptide (PACAP) are released at the RHT terminals (Hannibal, 2002) upon photic stimulation at night. They in turn bind to their receptors on the SCN neurons and evoke activation of intracellular signaling events that regulate clock gene expression and trigger clock resetting (Golombek and Rosenstein, 2010). At aforementioned, cellular rhythmic clock gene expression is driven by transcription/translational feedback loops. A major negative feedback loop is composed of CLOCK/BMAL1-driven rhythmic *Per* and *Cry* expression. *Per* and *Cry* levels are high during the day and low at night. Light at night triggers transient upregulation of *Per* and *Cry* expression, which will shift the phase of cyclic gene expression and reset the SCN clock. Light at the early night delays the clock whereas light at the late night advances the clock.

In searching for intracellular signaling pathways that mediate photic entrainment of the SCN clock, it is found that light at night activates S6K1 by inducing its phosphorylation at Thr389. In turn, activated S6K1 phosphorylates its downstream translation effectors including the ribosomal protein S6 (S6), a component of the 40S ribosomal subunit. S6K1 activation and S6 phosphorylation often correlate with translation efficiency of a subset of mRNAs which have a 5′-terminal oligopyrimidine (TOP) tract (Meyuhas and Dreazen, 2009), whereas evidence exists that neither S6K1 nor S6 phosphorylation is required for translational response of these mRNAs (Stolovich et al., 2002). In the SCN, it is found that protein products of TOP mRNAs such as *eEF1A* (eukaryotic elongation factor 1A) and *Jun B* are light-inducible in a rapamycin-sensitive manner (Cao et al., 2010). Light also increases phosphorylation of 4E-BP1 at Thr37/46 in the SCN (Cao et al., 2008). Phosphorylation of 4E-BP1 triggers its dissociation from eIF4E and activates cap-dependent translation. These activities are mTORC1-dependent, as rapamycin blocks light-induced S6K1 and 4E-BP1 phosphorylation. Light-induced mTORC1 activation appears to be important for photic entrainment of the SCN clock, as rapamycin modulates light-induced phase shifts of wheel-running and body temperature rhythms in mice (Cao et al., 2010). Effects of rapamycin on behavioral phase shift are consistent with its inhibition of light-induced PER1 and PER2 proteins in the SCN. Together, these results demonstrate that the mTORC1 signaling is an integral part of the photic entrainment pathway that regulates light-inducible mRNA translation in the SCN, although precise translational control mechanisms via S6K1 remain to be delineated.

**FIGURE 1:**
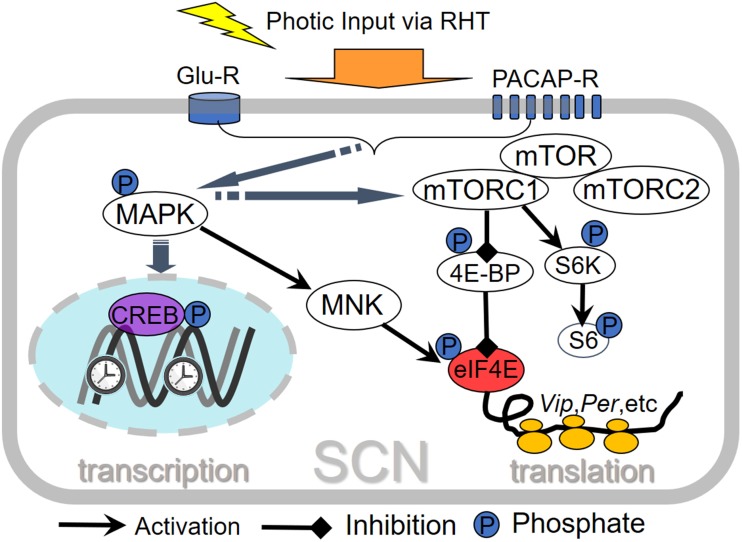
Schematic overview of light-regulated translational control pathways in the SCN. Light at night activates mTORC1, which in turn regulates its translational effectors ribosomal protein S6 kinases (S6Ks, including S6K1, and S6K2) and eukaryotic translation initiation factor 4E-binding proteins (4E-BPs). 4E-BP phosphorylation leads to its dissociation from eIF4E and activation of cap-dependent translational initiation. *Vip* (vasoactive intestinal peptide) mRNA translation is regulated by 4E-BPs. In another pathway, photic ERK/MAPK activation leads to phosphorylation of eIF4E via MNK kinases and promotes mRNA translation of *Per1* and *Per2* in the SCN. Thus, the MAPK and mTOR pathways converge on eIF4E to regulate mRNA translation in the SCN.

During photic entrainment, eIF4E is a pivotal point where the MAPK and mTORC1 pathways cross to control mRNA translation in the SCN. As aforementioned, MAPK pathway activates MNKs, which in turn phosphorylates eIF4E in the photo-recipient SCN cells and facilitates light-induced *Per1* and *Per2* mRNA translation. mTORC1 phosphorylates and inhibits the eIF4E repressor protein 4E-BP1. 4E-BP1 represses the eIF4E-dependent translation of *Vip*. Thus, mTORC1 activation disinhibits *Vip* mRNA translation and increases the abundance of *Vip* (also see section “Regulation of Synchronization of SCN Neurons by mTOR”). The light-regulated mTORC1 and MAPK pathways are summarized in **Figure 1**.

#### Regulation of Synchronization of SCN Neurons by mTOR

As the master pacemaker in mammals, unique anatomical and physiological features enable the SCN to generate accurate and robust rhythms. One of such features is the unique coupling mechanism among SCN neurons. SCN neurons are heterogenous in their expression of neuropeptides, pacemaking ability, response to light, and periods of their firing rhythms (Welsh et al., 1995, 2010; Herzog et al., 1998; Shirakawa et al., 2000; Aton and Herzog, 2005). In general, the ventral SCN neurons express VIP (vasoactive intestinal peptide), and the dorsal SCN neurons express AVP (arginine vasopressin). Some cells in between express GRP (gastrin releasing peptide). The ventral SCN cells receive photic input from the RHT and are directly entrained by light. In turn, these neurons send out output to the dorsal SCN neurons and reset their rhythms. To produce a coherent daily output, the SCN cells must entrain to each other. SCN intercellular coupling is essential for synchrony among cellular oscillators and robustness against genetic or environmental perturbations (Liu et al., 2007). Studies over the past decade have found that VIP signaling is particularly important for coupling SCN neurons.

Vasoactive intestinal peptide is a peptide of 28 amino acid and a ligand of G protein-coupled receptors (Gozes and Brenneman, 1993). *Vip* expression is enriched in the SCN cells and is also found in a subset of GABAergic neurons in the neocortex, olfactory bulb, some midbrain and brainstem regions as well as the gut and the pancreas (Liu et al., 2018). Through its receptor VPAC2R (encoded by the *Vipr2* gene), VIP signaling is essential for synchrony between ventral and dorsal SCN cells. Loss of VIP or VPAC2R leads to unstable, low amplitude circadian cycling in individual SCN cells and weak rhythms or arrhythmicity in SCN slices and animals (Harmar et al., 2002; Colwell et al., 2003; Cutler et al., 2003; Aton et al., 2005; Maywood et al., 2006). The direct protein product of the *Vip* gene is prepro-VIP, a 170-amino acid peptide. How *Vip* mRNA translation was regulated was not known.

As aforementioned, 4E-BPs are translational repressors and their activities are inhibited after phosphorylation by mTORC1. In the post-mitotic adult brain, cell growth and division are limited, and phosphorylation of 4E-BPs is low in a variety of brain regions, presumably because of the relatively moderate demand for protein synthesis. However, it is found that 4E-BPs are highly phosphorylated in the SCN (Cao and Obrietan, 2010), indicating a unique role for 4E-BPs in the SCN circadian clock. Indeed, it is found that 4E-BP1 specifically inhibits mRNA translation of *Vip*. By phosphorylating 4E-BP1, mTORC1 promotes *Vip* mRNA translation and increases the abundance of VIP in the SCN (Cao et al., 2013). In 4E-BP1 null mice, levels of prepro-VIP (precursor protein of VIP) and VIP are increased in the SCN. Consequently, these animals re-entrain to a shifted light/dark cycle more quickly and show resistance to the rhythm-disruptive effects of constant light. At the tissue level, the 4E-BP1 null SCN slices exhibit a shorter period and higher amplitude of PER2::LUCIFERASE (PER2::LUC) rhythms, consistent with enhanced coupling among SCN cells (Cao et al., 2013).

**FIGURE 2:**
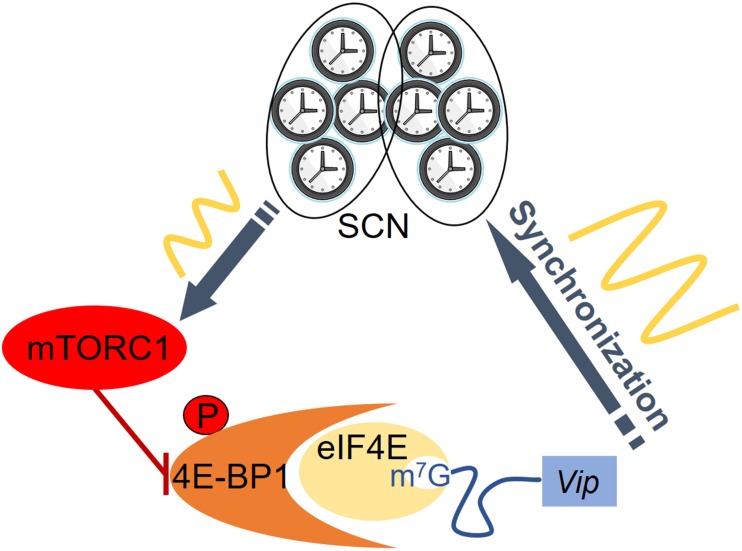
The mTORC1/4E-BP1 pathway regulates SCN cell synchrony via translational control of *Vip*. Rhythmic mTORC1 signaling phosphorylates 4E-BP1 to promote cap-dependent mRNA translation of *Vip* and increases the level of VIP in SCN. VIP signaling promotes synchrony of SCN cells and increases the robustness of clock gene oscillation. SCN clock synchrony can be enhanced by removing the translational repressor 4E-BP1.

Conversely, in *Mtor* heterozygotes prepro-VIP and VIP level is decreased, and the PER2::LUC rhythms in SCN are damped with a lengthened period (Cao et al., 2013; Ramanathan et al., 2018). These mice show longer period under constant conditions and are more susceptible to the effects of constant light (Cao et al., 2013). To test whether mTOR regulates SCN cell synchrony, the mTORC1 inhibitor PP242 was applied and PER2::LUC bioluminescence imaging was performed on SCN slices. SCN synchrony is indeed disrupted by PP242 (Liu et al., 2018), consistent with its rhythm-damping effects. As mTOR inhibition decreases *Vip* expression, the effects of PP242 may be ascribed to decreased VIP level in the SCN. To test whether mTOR regulates SCN synchrony through VIP neurons, conditional *mTOR* knockout mice were created, where *mTOR* gene was specifically knocked out in VIP cells (Liu et al., 2018). Indeed, these mice exhibit significant circadian defects, including weakened circadian behavioral rhythmicity under constant light, disrupted circadian behavior under a skeleton photoperiod, and decreased synchrony among SCN cells. These phenotypes largely resemble those seen in the *Vip* or *VPAC2* null mice (Harmar et al., 2002; Colwell et al., 2003; Cutler et al., 2003; Aton et al., 2005; Maywood et al., 2006) as well as in rats treated with VIP antagonists (Gozes et al., 1995), suggesting that mTOR regulates circadian synchrony via VIP signaling. However, additional mechanisms cannot be excluded. For example, as mTOR regulates the amplitude of cellular circadian oscillators (Ramanathan et al., 2018, see section “Regulation of Autonomous Properties of the Circadian Clocks by mTOR” ), decreased intercellular coupling could be due to attenuated cellular oscillations. Further studies are needed to identify additional mechanisms whereby the mTOR signaling controls SCN synchrony. **Figure 2** recapitulates the current model to explain how intracellular mTOR signaling can regulate intercellular coupling in the SCN via translational control of *Vip*.

#### Regulation of Autonomous Properties of the Circadian Clocks by mTOR

mTOR is a major integrator of intracellular signals that senses energy status (e.g., oxygen and ATP levels), and nutrients (e.g., leucine and arginine levels) to regulate cell growth and metabolism. Presumably it can serve as a linker between cellular metabolic states and the circadian timing process. A genome-wide RNAi screen in human cells identified hundreds of genes that regulate cellular clock functions (Zhang et al., 2009). The insulin signaling pathway was identified as the most overrepresented pathway. Downregulation of its multiple components such as PI3K and mTOR alters circadian period. Recently, the effects of mTOR manipulation on autonomous circadian clock properties were studied in various cellular and tissue oscillators (Ramanathan et al., 2018).

mTOR regulates fundamental clock properties (e.g., period and amplitude) in a variety of clock models. mTOR inhibition increases period and reduces amplitude, whereas activation of mTOR shortens period and augments amplitude in fibroblasts, hepatocytes, and adipocytes (Ramanathan et al., 2018). These results are consistent with studies showing dose-dependent lengthening of circadian period and damping of amplitude in human U2OS cells in response to rapamycin and torin1 treatments (Feeney et al., 2016; Lipton et al., 2017). Constitutive activation of mTOR in *Tsc2^−/−^* fibroblasts alters the dynamics of clock gene oscillations and elevates levels of core clock proteins, including CRY1, BMAL1, and CLOCK (Lipton et al., 2017; Ramanathan et al., 2018). However, serum stimulation upregulates CRY1 in an mTOR-dependent but *Bmal1*- and *Period*-independent manner (Ramanathan et al., 2018). Moreover, mTOR also regulates properties of the *ex vivo* SCN and liver clocks in a similar way (Ramanathan et al., 2018). In mice, heterozygous *mTor* knockout mice show lengthened circadian period of locomotor activity rhythms under constant conditions. Consistently, the 4E-BP1 knockout mice, where mTOR activity is increased, show shortened circadian period (Cao et al., 2013). However, TOR modulates circadian period in the opposite direction in *Drosophila*. Overexpressing S6K in the ventral lateral neurons, the central pacemaker cells, lengthens the circadian period (Zheng and Sehgal, 2010). Consistently, another study reports that knockout of *Tor* in *Per* expressing cells decreases circadian period of locomotor rhythms in flies (Kijak and Pyza, 2017). The reasons for this discrepancy between mice and flies are not clear, possibly due to different clock mechanisms in these species.

The circadian functions of mTOR in disease models are more intriguing. One study suggests that circadian rhythms of mTOR activities in cancer cells should be considered in chemotherapy In the study by Okazaki et al. (2014), circadian mTOR activities are found in mouse renal carcinoma. The rhythmic mTOR activities affect the efficacy of everolimus, a rapalog mTOR inhibitor that is clinically applied to treat cancers of the kidney, pancreas, breast, and brain. The drug is more effective in improving survival of tumor-bearing mice if applied at the time of a day when mTOR activities are elevated.

Studies also suggest that aberrant mTOR activities underlie circadian dysfunction under pathological conditions. In one study, mTOR is found to mediate the effects of circadian disruption caused by hypoxia, which is seen in many disease conditions such as cancer. When hypoxic cells are permitted to acidify to recapitulate the tumor microenvironment, the circadian clock is impacted through the transcriptional activities of hypoxia-inducible factors (HIFs) at clock genes. Acidification of cells suppresses mTORC1 signaling and restoring mTORC1 signaling rescues clock oscillation (Walton et al., 2018). In another study, Lipton et al. (2017) investigated circadian rhythms in a mouse model of TSC. In the TSC mice mTOR activities are constitutively elevated. They find that *Tsc*-deficient mice demonstrate shorter wheel-running period and disrupted core body temperature rhythms in constant darkness. Mechanistically, translation of *Bmal1* mRNA is increased and BMAL1 protein degradation is decreased, both of which lead to increased BMAL1 protein level and abnormal clock functions in the TSC tissues. Interestingly, reducing the dose of *Bmal1* genetically rescues circadian behavioral phenotypes in the TSC mouse models. The results, together with the findings of mTOR regulation of physiological clock properties, support a significant role for mTOR in circadian timekeeping under normal conditions as well as in mediating circadian dysfunction under disease conditions.

### Circadian Regulation of mTOR Activities and mRNA Translation

As is the case with many circadian clock-regulated signaling pathways, mTOR activities are regulated by the circadian clock and in turn the rhythmic mTOR activities reinforce the clock function. Indeed, one of the most prominent features of mTOR signaling is the temporal regulation of its activities by the circadian clock. Since mTOR was first studied in the SCN clock a decade ago, dozens of studies have identified circadian mTOR activities in different cells, tissues and organisms. First of all, mTORC1 activities exhibit robust circadian oscillations in the SCN under constant conditions, as indicated by rhythmic S6 and 4E-BP1 phosphorylation (Cao et al., 2011, 2013). In the mouse brain, mTORC1 activities also exhibit daily oscillations in the arcuate nucleus, hippocampus as well as the frontal cortex (Khapre et al., 2014; Saraf et al., 2014; Albert et al., 2015). These brain regions are important for circadian rhythms, feeding, learning, memory, and emotions. In *Drosophila*, TOR rhythms are also found in the brain and in particular the ventral lateral neurons (Zheng and Sehgal, 2010; Kijak and Pyza, 2017). In peripheral tissues, mTOR activities are rhythmic in the liver, cardiac and skeletal muscles, adipocytes, and retinal photoreceptors (Huang et al., 2013; Jouffe et al., 2013; Shavlakadze et al., 2013; Khapre et al., 2014; Drägert et al., 2015a,b; Lipton et al., 2015, 2017; Chang et al., 2016). Interestingly, mTOR also shows circadian rhythms in human osteosarcomas, mouse renal carcinomas as well as human breast cancer cells (Zhang et al., 2009, 2018; Okazaki et al., 2014). These circadian mTOR studies are summarized in **Table 1**. It remains elusive, however, what mechanisms drive rhythmic mTOR activities in different tissues and cells. In the SCN, cellular S6K1 activity levels correlates with cellular *Per1* but not *Per2* transcription due to unknown mechanisms (Cao et al., 2011).

**Table 1:** Circadian mTOR signaling in various tissues.

Tissue	mTOR cascade components	Temporal activity pattern	Regulation or function	Reference
Mouse SCN	S6K1, S6, 4E-BP1	Activated by light at early and late subjective night but not during the day	Modulates photic entrainment of SCN clock	Cao et al., 2008, 2010
Human osteosarcoma	mTOR	N.D.	Knockdown leads to longer circadian period	Zhang et al., 2009
*Drosophila* ventral lateral neurons	AKT, PTEN, RHEB, TSC, TOR, S6K	N.D.	Overexpression of TOR-S6K signaling lengthens circadian period; *Akt* hypomorphic mutants have a shorter circadian period	Zheng and Sehgal, 2010
Mouse SCN	S6	High at mid to late subjective day, peak at CT4-CT12	Colocalize with cellular *Per1* but not *Per2* transcription	Cao et al., 2011
Mouse liver	S6, 4E-BP1	High at night, low during day	Rhythmical translation of mRNAs involved in ribosome biogenesis	Jouffe et al., 2013
Mouse SCN	4E-BP1	High at late subjective day, early subjective night	Rhythmical translation of Vip mRNA, promoting SCN cell synchronization	Cao et al., 2013
Mouse skeletal muscles	S6, AKT	Low at ZT5, high at ZT21-ZT1	Regulated by fasting	Shavlakadze et al., 2013
Chicken retina	mTOR, S6K1, S6	High at late subjective day	Circadian regulation of protein level and functionality of L-type voltage-gated calcium channels in the retina	Huang et al., 2013
Mouse frontal cortex, heart	S6K1, S6	Frontal cortex: p-S6K1 is high at night Heart: p-S6 is high at late night and early day	N.D.	Khapre et al., 2014
Mouse renal cell carcinoma	mTOR, S6K1	High at night, low during day	Rhythmic mTOR activity influences the antitumor effect of mTOR inhibitors	Okazaki et al., 2014
Mouse hippocampus	4E-BP1, S6, AKT, mTOR	High during the day, low at night	Implicated in memory persistence	Saraf et al., 2014
Mouse liver and fibroblasts	S6K1, S6,	High at night, low during day	S6K1 rhythmically associates with and phosphorylates BMAL1 at S42; phosphorylation at Ser42 is important for its translational function	Lipton et al., 2015
Mouse adipose tissue	Rictor	Rictor and mTOR mRNA levels are high during the day and low at night	mRNA expression levels of core clock genes during the day are altered by Rictor conditional knockout; animals show higher blood pressure at night	Drägert et al., 2015a,b
Mouse arcuate nucleus	p-S6(Ser235/236), p-4E-BP1(Thr37/46); Raptor	High at early night, low at early day	Raptor knockout in agouti-related protein/neuropeptide Y (Agrp/NPY) neurons abolishes circadian expression of Agrp and NPY mRNAs	Albert et al., 2015
Rat cardiac and skeletal muscles	mTOR, S6K1	High during the day, low at night	N.D.	Chang et al., 2016
*Drosophila* brain	TOR	Highest at ZT20 and lowest at ZT4	Silencing of *TOR* in per expressing cells shortens circadian period of fly locomotor activity rhythms	Kijak and Pyza, 2017
Mouse brain and MEFs	TSC1 and TSC2	N.D.	mTOR regulates BMAL1 translation, degradation, and subcellular localization. Mouse models of TSC show high BMAL1 level and abnormal circadian rhythms	Lipton et al., 2017
Human breast cancer cell line MCF-7	p-S6(Ser240/244)	High from 12 to 24 h after serum shock	Delivery of Everolimus during 0∼24 h after serum shock induces a more evident G0/G1 blockage compared to delivery during 12∼36 h. Inhibition of mTOR activity reduces Cyclin D1 and Cyclin D3 protein levels	Zhang et al., 2018
Mouse SCN, liver, fibroblasts, hepatocytes, and lipocytes	mTOR, S6,4E-BP1	High at night, low during the day in liver	mTOR inhibition lengthens circadian period and damps amplitude, whereas mTOR activation shortens period and augments amplitude	Ramanathan et al., 2018
Neurospora crassa	NCU05950, homologous to the TOR pathway protein LAMTOR1 (mammals)	Arrhythmicity in NCU05950 protein levels	Anchored to the outer vacuolar membrane; mutation compromises FRQ-less rhythms	Ratnayake et al., 2018
Several human and mouse cell lines	mTORC1, p-4E-BP1, and p-S6K1	Rhythmic in normoxia buffer, amplitude, and phase changed in hypoxia buffer	Acidification of cells suppresses mTORC1 signaling and circadian rhythmicity. Restoring mTORC1 signaling by knockdown of 4E-BP1 partially rescues clock oscillation	Walton et al., 2018

Several mechanistic studies have highlighted a role for mTOR signaling as an output pathway which links circadian rhythmicity to mRNA translation. Jouffe et al. (2013) investigated circadian coordination of mRNA translation in the mouse liver. They identified rhythmic activation of a number of translational control signaling pathways, including the mTORC1 pathway and the ERK/MAPK pathway. They found that the circadian clock influences the temporal translation of a subset of mRNAs that are mainly involved in ribosome biogenesis. The circadian clock also controls the transcription of ribosomal protein mRNAs and ribosomal RNAs. Together these data demonstrate that the circadian clock exerts its function by temporal translation of a subset of mRNAs that are involved in ribosome biogenesis. In another study by the same group, they found that *Bmal1* deletion affects both transcriptional and post-transcriptional levels of rhythmic output. Translation efficiencies of genes with 5′-terminal oligopyrimidine tract (5′-TOP) sequences and genes involved in mitochondrial activity (many of which harbor a Translation Initiator of Short 5′-UTR motif) are differentially regulated during the diurnal cycle (Atger et al., 2015).

Lipton et al. (2015) made a surprising finding that the canonical clock protein BMAL1 also functions as a translation factor by associating with the translational machinery and promoting protein synthesis. Interestingly, translational activity of BMAL1 is regulated by rhythmic phosphorylation at Ser42 by the mTORC1/S6K1 pathway. S6K1-mediated phosphorylation is critical for BMAL1 stimulation of protein synthesis. Thus, these results demonstrate that the mTORC1/S6K1 pathway links circadian timing to rhythmic translation via BMAL1. Thus, the transcriptional feedback loop in the circadian clock is coupled to a translational regulatory loop mediated by the mTOR pathway. As translational control is involved in a number of cellular processes, this mechanism is potentially important to understand circadian regulation of many biological processes.

## Summary

mRNA translation is subject to complex regulation mechanisms. Among these, temporal regulation of mRNA translation occurs on a daily basis in various tissues as coordinated by the circadian clock and its output signaling pathways such as the mTOR signaling. In turn, rhythmic mTOR signaling and mRNA translation feedback to the clock machinery and regulate important clock functions, including its timing, response to entrainment cues, as well as the network properties among circadian oscillators. Deregulation of translational control is linked to circadian clock dysfunction, as seen in the TSC and hypoxia mouse models. Knowledge of mTOR and translational control in the circadian clock is not only essential for understanding the basic clockwork mechanisms, but also could provide insights into mechanistic links between circadian dysfunctions and human diseases so that therapeutic strategies can be developed for these disorders.

## Author Contributions

The author confirms being the sole contributor of this work and approved it for publication.

## Conflict of Interest Statement

The author declares that the research was conducted in the absence of any commercial or financial relationships that could be construed as a potential conflict of interest.
